# Immunotherapy and stereotactic body radiotherapy for older patients with non-metastatic renal cancer unfit for surgery or decline nephrectomy: practical proposal by the International Geriatric Radiotherapy Group

**DOI:** 10.3389/fonc.2024.1391464

**Published:** 2024-05-24

**Authors:** Nam P. Nguyen, Monica-Emilia Chirila, Brandi R. Page, Vincent Vinh-Hung, Olena Gorobets, Mohammad Mohammadianpanah, Huan Giap, Meritxell Arenas, Marta Bonet, Pedro Carlos Lara, Lyndon Kim, Fabien Dutheil, David Lehrman, Luis Zegarra Montes, Ghassen Tlili, Zineb Dahbi, Gokoulakrichenane Loganadane, Sergio Calleja Blanco, Satya Bose, Elena Natoli, Eric Li, Abba Mallum, Alessio G. Morganti

**Affiliations:** ^1^ Department of Radiation Oncology, Howard University, Washington, DC, United States; ^2^ Department of Clinical Development, MVision AI, Helsinki, Finland; ^3^ Department of Radiation Oncology, Amethyst Radiotherapy Centre, Cluj-Napoca, Romania; ^4^ Department of Radiation Oncology, Johns Hopkins University, Baltimore, MD, United States; ^5^ Department of Radiation Oncology, Centre Hospitalier Public du Contentin, Cherbourg-en-Contentin, France; ^6^ Department of Oral Surgery, University Hospital of Martinique, Fort-de-France, France; ^7^ Colorectal Research Center, Department of Radiation Oncology, Shiraz University of Medical Sciences, Shiraz, Iran; ^8^ Department of Radiation Oncology, Medical University of South Carolina, Charleston, SC, United States; ^9^ Department of Radiation Oncology, Sant Joan de Reus University Hospital, University of Rovira, I Virgili, Tarragona, Spain; ^10^ Department of Radiation Oncology, Arnau de Vilanova University Hospital, Lleida, Spain; ^11^ Department of Radiation Oncology, Fernando Pessoria Canarias Las Palmas University, Las Palmas, Spain; ^12^ Division of Neuro-Oncology, Mount Sinai Hospital, New York, NY, United States; ^13^ Department of Radiation Oncology, Clinique Sainte Clotilde, Saint-Denis, Reunion Island, France; ^14^ Department of Radiation Oncology, International Geriatric Radiotherapy Group, Washington, DC, United States; ^15^ Department of Urology, University of Peruana, Lima, Peru; ^16^ Department of Urology, Sahloul University Hospital, Sousse, Tunisia; ^17^ Department of Radiation Oncology, Mohammed VI University of Health Sciences, Casablanca, Morocco; ^18^ Department of Radiation Oncology, Institut Curie, Paris, France; ^19^ Department of Oral Maxillofacial Surgery, Howard University, Washington, DC, United States; ^20^ Department of Radiation Oncology, Istituto di Ricovero e Cura a Carattere Scientifico (IRCCS) Azienda Ospedaliera-Universitaria di Bologna, Bologna, Italy; ^21^ Radiation Oncology, Department of Medical and Surgical Sciences (DIMEC), Alma Mater Studorium, Bologna University, Bologna, Italy; ^22^ Department of Pathology, Howard University, Washington, DC, United States; ^23^ Department of Radiation Oncology, University of KwaZulu Natal, Durban, South Africa

**Keywords:** older, renal cancer, CPI, SBRT, protocol

## Abstract

The standard of care for non-metastatic renal cancer is surgical resection followed by adjuvant therapy for those at high risk for recurrences. However, for older patients, surgery may not be an option due to the high risk of complications which may result in death. In the past renal cancer was considered to be radio-resistant, and required a higher dose of radiation leading to excessive complications secondary to damage of the normal organs surrounding the cancer. Advances in radiotherapy technique such as stereotactic body radiotherapy (SBRT) has led to the delivery of a tumoricidal dose of radiation with minimal damage to the normal tissue. Excellent local control and survival have been reported for selective patients with small tumors following SBRT. However, for patients with poor prognostic factors such as large tumor size and aggressive histology, there was a higher rate of loco-regional recurrences and distant metastases. Those tumors frequently carry program death ligand 1 (PD-L1) which makes them an ideal target for immunotherapy with check point inhibitors (CPI). Given the synergy between radiotherapy and immunotherapy, we propose an algorithm combining CPI and SBRT for older patients with non-metastatic renal cancer who are not candidates for surgical resection or decline nephrectomy.

## Introduction

The management of renal cancer remains a challenge for older patients. Surgical resection is the standard treatment for non-metastatic renal cancer. However, due to the presence of co-morbidities, older patients may not benefit from surgery. In a study of 537 patients aged 75 or above with localized renal cancer 7 cm in size or less, nephrectomy has led to a poor survival as patients died from cardiovascular disease and deterioration of renal function in the remaining kidney (1). Compared to radical nephrectomy, a partial nephrectomy for localized renal cancer may better preserve renal function but did not improved survival among patients aged 65 or older (2). Regardless of age or type of surgery, frail patients with renal cancer are at increased risk for major complications and poor survival after the procedure (3, 4). Preserving renal function is imperative for averting the necessity of dialysis, mitigating chronic kidney disease, and reducing mortality associated with cardiac events (5). Thus, older and frail renal cancer patients may not be candidates for surgery and need an alternative for curative treatment when diagnosed at an early stage.

In the past, renal cancer was considered to be radio-resistant and required a higher dose per fraction (hypofractionation) in order to overcome the tumor cell ability to repair radiation damage (6). However, the delivery of a high radiation dose may also lead to serious complications due to damage to the normal organs at risk (OAR) surrounding the cancer with older radiotherapy techniques. The introduction of stereotactic body radiotherapy (SBRT) in the treatment of early stage non-small cell lung cancer (NSCLL) has led to its successful application for non-metastatic renal cancer in patients who are not surgical candidates due to their age and pre-existing comorbidities (7). Preliminary studies are very promising with excellent local control and survival in selected patients with small tumors and low grade histology (8, 9). As a local treatment similar to surgery, SBRT for renal cancer may not be effective for tumors with high risk for loco-regional recurrences and distant metastases due to their size and aggressive histology. Those tumors often carry program death ligand 1 (PD-L1) which allow them to evade immune surveillance and make them an ideal target for immunotherapy with checkpoint inhibitors (CPI) (10, 11). As high dose radiotherapy has a synergistic effect with CPI, this combination may be ideal for the treatment of older cancer patients with non-metastatic renal cell cancer (12).

The International Geriatric Radiotherapy Group (http://www.igrg.org) is an organization devoted to the care of older cancer patients, minorities, and women who are frequently excluded from clinical trials (13). Based on currently published literature, members of the genitourinary cancers subgroup propose in this article a practical protocol for older patients with non-metastatic renal cancer who are too frail to undergo surgery or who decline nephrectomy. Radiotherapy and immunotherapy may induce long-term remission and potential cure for those patients.

## Rationale for using immunotherapy in renal cancer

### Renal cancer immune environment

Renal cell cancer has a very complex tumor immune microenvironment (TIME) which depends on the tumor histology and evolves over the course of treatment, thus defying any simple classification (14–17). Most studies have focused on clear cell renal (CCR) carcinoma which comprises the majority (up to 80%) of the tumor subtypes. Other non-CCR carcinoma such as the sarcomatoid subtype may have a more aggressive biology behavior and a different TIME (18). In general, renal cell TIME is characterized by an overwhelming abundance of immunosuppression which allows cancer cells to evade immune surveillance and cause disease progression. A preponderance of immunosuppressive cytokines such as interleukin-10 (IL-10) and transforming growth factor beta (TGF-β) promotes the differentiation of regulatory T cells (Treg) which in turn inhibit CD8+ T cells from tumor killing (19–25). In addition, tumor cells may also express PD-L1 which binds with the programmed cell death protein 1 (PD-1) on CD8+ T cells to neutralize its tumoricidal function (10, 11). Hypoxia is also another contributing factor to the tumor immune resistance (26, 27). Thus, any effective treatment should target all of the elements that contribute to the tumor ability to evade immunosurveillance. Even though PD-L1 is not a perfect biomarker, increase in PD-L1 expression has been reported to be correlated with a poor prognosis. Among 346 patients with renal cell carcinoma who had long-term follow-up, high PD-L1 expression was correlated with increased tumor size, high nucleolar grade, lymph nodes invasion, tumor recurrence, and cancer specific death, and sarcomatoid subtype (28, 29). The adverse histopathological features linked to PD-L1 expression has been corroborated in other studies for clear cell and non-clear cell renal carcinoma (30, 31). In another study of 381 patients with renal cell carcinoma who underwent nephrectomy, 120 patients (31.4%) had PD-L1 in the surgical specimen. Compared to patients who were PD-L1 negative, those with positive biomarker had shorter time to recurrence and decreased survival (32). Two meta-analysis also corroborated the poor prognosis conferred by PD-L1 expression in renal cell carcinoma for early disease stage and for patients with distant metastases (10, 33). Conversely, PD-L1 expression is also associated with an excellent response to immunotherapy with CPI (34). Thus, any induced increase in PD-L1 expression in renal cell carcinoma may lead to an improved response to immunotherapy and potentially better survival.

### Alteration of renal cancer immune environment with radiotherapy

Radiotherapy produces a significant alteration of the renal TIME which is dose dependent and not fully understood. At high dose level, hypofractionated radiotherapy predominantly produces a pro-immunogenic tumor environment through endothelial cell apoptosis induced by activation of ceramide which in turn initiates the release of mitochondrial cytochrome c (35–37). As renal cell cancer is a hypervascular tumor, this may account for tumor shrinkage following SBRT (38, 39). In addition, there is significant infiltration of CD8+T cells in the tumor microenvironment after an ablative dose of radiation leading to eradication of the primary tumor (40). The role of T cells-induced by radiation is highlighted in a study of early stage renal cancer treated with SBRT to a total dose of 15 Gy followed by nephrectomy four weeks later. A significant infiltration of T cells was observed not only in the surgical specimen but also in the bloodstream of patients receiving preoperative radiotherapy compared to the ones who had surgery alone (41). Increased of T cells in the tumor microenvironment is postulated through the production of interferon gamma (IFNγ) by inflammatory cells (T helper 1, natural killer, and natural killer T cells) following radiation (34). However, increased in IFNγ production may also lead to an increase in PD-L1 expression by the tumor cells which may attenuate the immune response as the cancer cells may escape killing by CD8+T cells (42–44). The dual role of IFNγ may explain the upregulation of PD-L1 expression in many solid tumors following radiotherapy and confers resistance to the immune effect of radiotherapy. The increase in PD-L1 expression may also serve as a strategy for clinicians to combine radiotherapy and CPI to improve survival of patients with renal cancer (45, 46).

## Effectiveness of immunotherapy for renal cancer

### The role of CPI for resectable renal cancer

Even though surgical resection remains the treatment of choice for early stage renal cancer, up to 30% of the patients may develop loco-regional recurrence and/or distant metastases following the procedure. Many algorithms have been proposed to assess the recurrence risk for those patients based on tumor size, histologic grade, histology subtype such as sarcomatoid histology, and pathological stage (47–49). Thus, an attempt is made to use neoadjuvant immunotherapy to reduce recurrence risks for high risk renal cancer patients. The rationale for neoadjuvant immunotherapy relies on its theoretical ability to improve immune surveillance, thus reducing the risk of micrometastases.

Preliminary experience for neoadjuvant immunotherapy has been promising with minimal serious side-effects during the neoadjuvant phase and acceptable surgical complications (50–55). Three studies investigated the response of non-metastatic renal carcinoma to nivolumab after three to four cycles. There was an intense infiltration of CD8+ T cells in the surgical specimen even although the tumor size remained mostly stable (50–52). Two other studies included patients with metastatic disease who underwent nephrectomy following neoadjuvant immunotherapy with a combination of CPI and other biologic agents (53, 54). Interestingly, 13% of the patients achieved a complete pathologic response in the primary renal cancer (53). However, only one study had PD-L1 investigated in the initial biopsy (7% positivity rate) (54). Thus, the correlation between PD-L1 positivity and response rate to CPI remains to be investigated in future studies. Patients who had a high CD8+T cells in the biopsy specimen may have a better response and improved survival. Those studies are limited by the small number of patients and a short follow-up. However, they illustrated that CPI are well tolerated and do not impair the surgical outcome. **Table 1** summarizes relevant studies on the use of neoadjuvant immunotherapy for renal cell cancer.

**Table 1:** Relevant studies on the use of neoadjuvant immunotherapy for renal cell carcinoma.

Study	Patient No.	Biologic agent	Response rate	Recurrence	Survival	Complications
Gorin et al. (50)	17	Nivo	Stable	11.7%	85.7% (3-year)	11.8% gr.3
Carlo et al. (51)	18	Nivo	15%	18%	NS	11% gr.3
						11% postoperative complications
Singla et al. (52)	15	Nivo	Stable	NS	NS	NS
Panian et al. (53)	52	Various	42%	36.3%	NS	None
Alex et al. (54)	40	Avelumab	30%	32%	NS	20% gr 3
		Axitinib				

Nivo, Nivolumab; Gr, grade; NS, not specified.

Among patients at high risk for recurrence after nephrectomy for renal cancer, pembrolizumab given every three weeks up to one year has been reported to improve recurrence rates and disease-free survival (DFS) compared to patients who received placebo (55). Recurrence rates were 22% and 33% for pembrolizumab and placebo, respectively. Corresponding distant metastases rates were 22.7% and 31.2%, respectively. At 30 months follow-up, DFS was 70.6% and 64.8% for pembrolizumab and placebo, respectively. In the third interim analysis, there was also a 38% reduction of death with adjuvant pembrolizumab compared to placebo at a follow-up of 48 months (56). There was no difference in outcome between patients who were PD-L1 positive or negative. However, there was a surprisingly high proportion of PD-L1 positive patients in both groups, 74% and 77% for pembrolizumab and placebo, respectively, which may have accounted for the benefit of pembrolizumab in the adjuvant setting. The positive outcome of immunotherapy for high risk renal cancer after nephrectomy has not been corroborated in two other trials with atezolizumab and nizolumab combined with ipilimumab (57, 58). In the adjuvant atezolizumab trial, 778 renal cancer patients with high risk of recurrence after surgery was randomized between atezolizumab (n=390) every three weeks for one year or placebo (n=388). T. Median DFS was 57.2 months and 47.9 months for patients receiving atezolizumab and placebo, respectively (57). Thus, atezolizumab did not improve the clinical outcome. However, compared to the study with adjuvant pembrolizumab, the proportion of patients with PD-L1 expression was lower and may have accounted for the survival difference. The proportion with positive PD-L1 was 59% and 61% for the atezolizumab and placebo arms, respectively. In the study comparing the combination of nivolumab and ipilimumab to placebo, 816 patients was randomized to both CPI (n=405) or placebo (n=411) after surgery for renal cell carcinoma with high risk features. There was no difference in DFS between these two groups (58). However, PD-L1 was not investigated as a biomarker, thus, many questions remain unanswered about the efficacy of CPI for patients at high risk for recurrence after nephrectomy for renal cancer. It is clear that the influence of PD-L1 as a biomarker for CPI efficacy should be investigated in future prospective studies of renal cell cancer.

Recently, a novel and potent immune indicator for predicting immunotherapy response and oncology outcomes has been proposed for solid tumors. Immunoscore is based on immunohistochemistry and quantitative measurement of the density of CD3+ and CD8+ cytotoxic T cells in two different locations of the tumor center and the margin of tumor invasion. Intermediate and high immunoscore predict favorable response to immunotherapy and good prognosis (59). Preliminary studies suggest a powerful predicting and prognostic role for this scoring system in renal cell carcinoma (60, 61). Thus, immunoscore could be part of a protocol study on immunotherapy for renal cell cancer.

### The role of CPI for advanced or metastatic renal cancer

In contrast to the controversy surrounding immunotherapy for resectable cancer at risk for recurrence after nephrectomy, the combination of CPIs or a CPI with an anti-vascular endothelial growth factor (VEGF) antibody or tyrosine kinase inhibitor (TKI) has become the standard of care for metastatic renal cancer (62).

Nivolumab and ipililumab have been reported to have superior survival and DFS compared to sunitinib for advanced renal carcinoma with a clear cell component (63). The 4-year survival was 53.4% and 43.3% for nivolumab and ipililumab, and sunitinib, respectively. In another study with a similar population of renal cancer patients, avelumab (PD-L1 antibody) and axitinib, an anti-VEGF TKI also demonstrated superior progression-free survival (PFS) compared to sunitinib. The median PFS at 13 months was 13.3 and 8 months for avelumab and axitinib and sunitinib, respectively (64). Corresponding numbers for patients with positive PD-L1 tumors, was 13.8 and 7 months for the combination group, and sunitinib, respectively. Thus, patients who had PD-L1 expression had a better outcome when treated with CPI. Another anti PD-1 agent, pembrolizumab was also effective when combined with axitinib for the treatment of advanced clear cell carcinoma (65, 66). At a median follow-up of 42 months, the survival rate was 57.5% and 48.5%, for the combination arm, and sunitinib, respectively (65). Other studies also demonstrated the superiority of combining immunotherapy and an anti-VEGF agent compared to sunitinib: Nivolumab and cabozantinib, pembrolizumab and lenvatinib (67, 68). However, it is unclear which combination is most effective for those patients even though the highest complete response (CR) rate (16%) has been reported with the lenvatinib combination (68).

Taken together, given the complex immune micro-environment of renal cancer, a combination treatment with immunotherapy and another agent may be more effective than immunotherapy alone to overcome the tumor ability to evade killing by the immune system. Radiotherapy may potentially further improve survival and loco-regional control for those patients due to its synergy with immunotherapy, if excessive irradiation to the normal organs could be avoided.

### Efficacy of immunotherapy among older cancer patients with renal cancer

A meta-analysis of studies using immunotherapy alone or combined with other anti-VEGF agents as first-line of treatment demonstrated that older patients with renal cancer defined as 65 years of age or older had improved survival compared to the ones receiving sunitinib (69). Again, the combination of lenvatinib and pembrolizumab seems to be most promising but needs to be confirmed in future prospective studies (69). There was no difference in survival among patients 75 years of age or older compared to other younger age groups who received immunotherapy for metastatic renal cancer (70). However, they may be more prone for dose reduction to minimize treatment toxicity due to their frailty status (70). Thus, older renal cancer patients receiving immunotherapy should be monitored closely by a team familiar with geriatric care. Other studies also corroborated the safety profile of immunotherapy for older patients with other solid tumors such as bladder cancer (71–74).

## The role of SBRT in the management of non-metastatic renal cancer

The combination of intensity-modulated radiotherapy with precision image-guidance has brought a new era in the treatment of cancer thought to be radio-resistant such as renal cell cancer and melanoma. Daily imaging before treatment allows delivery of a high dose of radiation to the target while minimizing damage to the OAR, thus improving local control and potential cure for localized disease. Serious side effects and complications are significantly reduced to allow frail patients who are not candidates for surgical resection to have an alternate treatment option with curative intent. As an illustration, older NSCLC patients with early disease stage had an excellent local control and survival following SBRT (7).

Even though other non-surgical treatment modalities for renal cancer are available such as cryotherapy or microwave ablation, they are limited by the size of the tumor, the proximity of the ureters and the large vessels in patients who may also require anticoagulants due to the tumor thrombus (75). Excellent local control may be achieved with large renal cancers (median 4.9 cm) treated with SBRT even though those tumors frequently develop distant metastases after treatment and may be candidates for systemic therapy (76). The tumor shrinks slowly over time after SBRT and the irradiated kidney develops atrophy proportional to the radiation dose (77). However, even though the ipsilateral kidney function deteriorated over time after treatment, the spared contralateral kidney function may improve and allow a better renal function preservation (78). In a prospective pilot study, Kirste et al. (79) applied SBRT using five fractions of 10 Gy or eight fractions of 7.5 Gy for the treatment of seven patients with renal cancer who were affected with the Von Hippel-Lindau disease. The patient tolerated SBRT well and no patient experienced acute or chronic grade 2 or more toxicity. After a median follow-up of 43 months, the 2-year locol control and cancer-specific survival were 100% with long-term renal preservation. As older patients renal function usually decreases over time, SBRT may be the best suited treatment option for those patients with unresectable or medically inoperable cancer (80). In addition, compared to other non-surgical procedures such as radiofrequency ablation (RFA), it is technically much easier to perform SBRT. As an illustration, in a trial which was initially designed to compare the efficacy between SBRT and RFA for small size renal cancer, 24 patients were recruited with the intent to have 12 patients in each arm. However, after randomization, only 7 was assigned to RFA due to the technical difficulty to perform the procedure. Two other patients was reassigned to SBRT, and three refused any procedure. Even though there was an imbalance between the two arms, there was no difference in survival between the two groups which highlights the effectiveness of SBRT for renal cancers (81).

Even though SBRT is a safe procedure with preliminary excellent outcome, many questions remain unanswered as each institution has different protocols for the dose fractionation and techniques of irradiation. In addition, a national survey of stage I renal cell carcinoma treated with different modalities, suggests that SBRT may have an inferior survival outcome compared to partial nephrectomy or thermal ablation (82). However, SBRT was performed in non-academic centers which may have less experience in treating renal carcinoma. Survey of centers with SBRT expertise in treating a large number of renal cancers reported excellent local control and survival.

Siva et al. (83) reported a prospective non-randomized trial of 70 patients from eight institutions with biopsy proven renal carcinoma and a median size of 4.6 cm (range 3.7 to 5.5 cm) treated with SBRT (FASTRACT II trial). The dose ranged from 26 Gy single fraction (<4 cm) or 14 Gy times three (>4 cm). At a median follow-up of 42 months, local control and survival was 100%. Only 10% developed grade 3 complications. Thus, in a well-designed multi-institution study with selected patients and strict protocol enforcement, SBRT is safe and effective. Other studies also corroborated the excellent local control achieved with SBRT for small tumors (4 cm or less) with minimal complications ranging from 0 to 10% depending from the length of follow-up (84–86). For example, in a meta-analysis of 190 patients treated with SBRT for renal carcinoma with either single or multiple fractions from the IROCK (the International Radiosurgery Consortium of the Kidney), local control was 94.5% at 5 years (86). However, similar to reports from surgical studies for non-metastatic renal carcinoma, size of the tumor remains a poor prognostic factor. The maximum size of tumor is a significant predicting factor of death linked to the development of distant metastases (76, 85). Thus, a treatment strategy needs to be developed for those patients to improve their survival. In addition, other poor prognostic factors such as tumor grade and sarcomatoid subtype need to be investigated in future prospective SBRT studies.

Preliminary report suggests that patients with renal cell carcinoma may enjoy a good quality of life (QOL) following SBRT despite the fact that many are old and have co-morbidity factors that preclude them from having surgery. Swaminath et al. (87) reported the QOL of 28 patients who underwent SBRT for renal cell carcinoma with the Functional Assessment of Cancer Therapy-Kidney Symptoms Index-19 (FACT FKSI-19) and the European Organization for Research and Treatment of Cancer Quality of Life Questionnaire Core-15 Palliative (EORTC-QLQ-C15-PAL). There was little change of QOL over time from the baseline prior to treatment and six months after SBRT. Interestingly, emotional score improves over time likely related to the significant decrease of pain produced by the reduction in size of the tumor mass. As kidney cancer becomes atrophic and shrinks over time, it is anticipated that their QOL may further improve with long-term follow-up (77). However, further studies should be performed to verify this hypothesis.

There is still a debate about the optimum dose selection for the treatment of renal cancer with SBRT. Small tumors (4 cm or less) tend to be treated with a single fraction which may be more convenient for older patients with transportation difficulty. Larger tumors are frequently treated with multiple fractions ranging from three to ten. However, most institutions use a protocol of three to five fractions for patient and staff convenience. Many institutions have performed phase I dose escalation study to assess what is the maximum dose that may be achieved without having excessive toxicity (88, 89). An alternative question would be about the biologic equivalent dose (BED) necessary to control tumors of different sizes. Kurban et al. (90) reported the pathology of 323 nephrectomies for renal mass with tumor size ranging from 4 cm or less (small), 4 to 7 cm (intermediate), and greater than 7 cm (large). Ninety percent of the small tumors were localized to the kidney and were of low histologic grade. Large tumors often invaded adjacent tissues, and presented with aggressive features such as high grade, necrosis, and sarcomatoid changes. Thus, it would be easy to eradicate a small tumor with a single fraction of 26 Gy for example. Hypoxia and necrosis associated with larger tumors often confer radio-resistance and may require a higher BED to overcome their resistance. Even though there is still debate on the value of the α/β value for renal cancer, Tran et al. (91) using an α/β ratio of 3 to review the literature on SBRT for renal cancer suggests that a BED3 of 225 or more which corresponds to 48 to 60 Gy in 3 fractions or 48 Gy in 4 fractions may be associated with a better survival.

Thus, for large tumors either a high BED or combining SBRT with a radiosensitizing agent such as CPI may improve local control and/or survival. The combination of immunotherapy and SBRT may be more attractive due to the potential to eradicate micrometastases and survival. As a local therapy like nephrectomy, SBRT would not impact the development of distant metastases in tumors with high risk features for recurrence.

## Safety profile of immunotherapy and hypofractionated radiotherapy for advanced or metastatic renal carcinoma

Due to the synergy between immunotherapy and radiotherapy for renal cancer, and in particular the potential beneficial effect of the radiotherapy-induced abscopal effect, many institutions have conducted trials to assess the feasibility of SBRT or radiosurgery with immunotherapy for metastatic disease (34, 92–98).

Preliminary results are very promising. The combination of hypofractionated radiotherapy and immunotherapy is safe. There is no reported treatment related death (34, 92–97). Grade 3–4 toxicity ranged from 5.6 to 30%. Selected studies suggest a survival advantage combining radiotherapy and immunotherapy versus immunotherapy alone for metastatic renal cancer.

Piening et al. (96) reported the survival outcome of 644 patients with metastatic renal cancer who received hypofractionated radiotherapy combined with CPI (n=63) or CPI alone (n=581). The 2-year survival for patients with brain metastases was significantly improved for the combined therapy, and was 70.8% and 51.4% for the radiotherapy with CPI arm and CPI alone, respectively. Timing of immunotherapy before or after radiotherapy had no impact on survival. Even though that was a retrospective study, the benefit of adding radiotherapy to CPI is also corroborated in other trials (94, 97, 98). For example, Li et al. (98) reported in a randomized study the benefit of adding a split course of radiotherapy to nivolumab (n=22) compared to nivolumab alone (n=22). Even though the patient number is small, median PFS was 28.1 and 21.5 months for the combined modality and nivolumab alone, respectively. Patients with oligometastases seem to benefit the most from the combined treatment.

Siva et al. (97) treated 30 renal cancer patients with one to five metastatic sites with a single course of 20 Gy SBRT or 30 Gy in 10 fractions to all metastatic sites followed by pembrolizumab 200 mg administered every three weeks for eight cycles. At a median follow-up of 28 months, 2 year survival and disease control rate was 74% and 83%, respectively. In another study, Li et al. (98) reported the outcome of 44 patients with renal oligo metastases randomized to immunotherapy alone (n=22) or combined with radiotherapy (n=22) at a dose of 50 Gy in 5 fractions. The objective response rate was 59% and 27% for the combined treatment and immunotherapy alone, respectively. Corresponding numbers for progression-free survival was 28.1 and 21.5 months respectively. There was no difference in adverse events between those two groups. Thus, immunotherapy is safe and may be effective in selected patients when combined with high dose radiation. However, the caveat of those studies is the lack of biomarkers such as PD-L1 to assess response rate and survival. They did highlight the fact that immunotherapy can be safely integrated in a protocol using SBRT for non-metastatic renal carcinoma in patients with high risk features for recurrences.

## Evaluation of frailty in older patients with renal cell carcinoma

Before enrolling any older cancer patients (defined as 65 years old or above) in any protocol, frailty needs to be assessed due to its impact on the treatment. Frailty is defined as a state of increased vulnerability resulting from aging associated decline in reserve and function across multiple physiologic systems (99). Even though there are many questionnaires to assess frailty in older patients. the G-8 questionnaire is simple to administer in a busy clinic, thus practical to implement in clinical trials (100). Those with a score of 15 or above are defined as fit. Those with a score of 14 or less will undergo a complete geriatric assessment with the comprehensive geriatric assessment (CGA) survey (101). Thus, any impact of frailty on patient tolerance to treatment could be recorded and be used to develop future treatment protocols on the combination of immunotherapy and radiotherapy for renal cancers. In addition, to achieve optimal technical outcome in older cancer patients who may have mental issue in collaborating with immobilization protocols such as 3D exhale breath-hold technique, cognitive assessment questionnaire such as Mini-Mental Status Exam (MMSE) or Montreal Cognitive Assessment (MoCA) may be useful to assess their suitability for collaboration (102).

## Proposed IGRG algorithm for older patients with non-metastatic renal cancer who are not candidates for surgery or decline surgery

All tumor biopsy specimen should undergo next generation sequencing (NGS) if feasible which includes PD-L1 and other potential biomarkers for immune response. However, if NGS is not feasible, PD-L1 status should be confirmed with immunohistochemistry. All patients should be assessed for frailty prior to their enrollment to investigate its impact on the combined treatment. Patients with small (4 cm or less) cancers of low grade histology and non-aggressive subtypes should undergo SBRT alone to a dose of 26 Gy single fraction as they are likely to have excellent local control and survival. Immunotherapy is unlikely to add any benefit for those patients but could be used for salvage therapy in case of recurrence. Patients with large tumors (more than 4 cm) and/or associated with high risk for recurrences such as high grade histology or aggressive subtypes should be stratified based on their PD-L1 status. Those with PD-L1 with 1% or more should undergo immunotherapy first for four cycles before radiotherapy as they are likely to respond to CPI. They should undergo fractionated SBRT to achieve a BED3 of 225 Gy (91). Immunotherapy should be resumed for four cycles after SBRT unless the patient developed significant toxicity to CPI during the induction phase to achieve a total of eight cycles (97).

Those with PD-L1 less than 1% should receive SBRT first with the same fractionation and BED to induce upregulation of PD-L1 followed by eight cycles of immunotherapy unless they develop undue toxicity to CPI. We postulate that the combination of immunotherapy and SBRT may improve survival for those patients as it may decrease the risk of micrometastases and improve local control in large tumors which are often necrotic and hypoxic.

**Figure 1:**
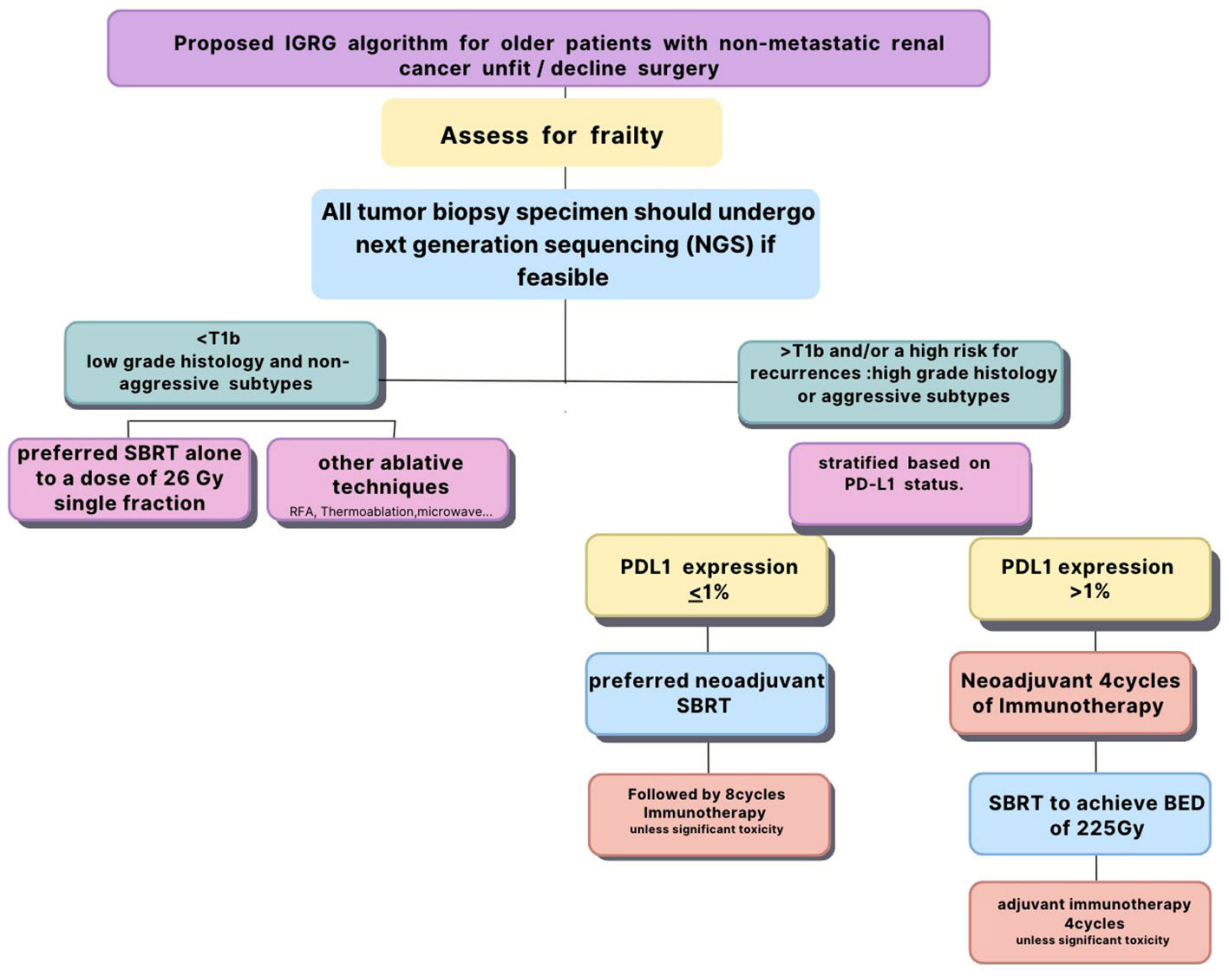
International Geriatric Radiotherapy protocol for non-metastatic renal cancer.

The conclusions based on prospectively collected data may improve the design of future clinical trials targeting older patients treated with immunotherapy and SBRT for renal cancer. **Figure 1** summarizes the proposed algorithm.

With a network of 1282 cancer institutions across the world and a large number of patients from all ethnicities, the IGRG is committed to conduct those studies when funding becomes available (103, 104).

## Conclusion

The combination of SBRT and immunotherapy may be beneficial for older patients with non-metastatic renal cancer who are not candidates for surgical resection or decline nephrectomy. Prospective studies should be conducted to verify this hypothesis.

## Data availability statement

The original contributions presented in the study are included in the article/supplementary material. Further inquiries can be directed to the corresponding author.

## Author contributions

NN: Writing – original draft, Writing – review & editing. M-EC: Writing – original draft, Writing – review & editing. BP: Writing – original draft, Writing – review & editing. VV-H: Writing – original draft, Writing – review & editing. OG: Writing – original draft, Writing – review & editing. MM: Writing – original draft, Writing – review & editing. HG: Writing – original draft, Writing – review & editing. MA: Writing – original draft, Writing – review & editing. MB: Writing – original draft, Writing – review & editing. PL: Writing – original draft, Writing – review & editing. LK: Writing – original draft, Writing – review & editing. FD: Writing – original draft, Writing – review & editing. DL: Writing – original draft, Writing – review & editing. LM: Writing – original draft, Writing – review & editing. GT: Writing – original draft, Writing – review & editing. ZD: Writing – original draft, Writing – review & editing. GL: Writing – original draft, Writing – review & editing. SCB: Writing – original draft, Writing – review & editing. SRB: Writing – original draft, Writing – review & editing. EN: Writing – original draft, Writing – review & editing. EL: Writing – original draft, Writing – review & editing. AAM: Writing – original draft, Writing – review & editing. AGM: Writing – original draft, Writing – review & editing.
